# Complete reference genomes of two ceftriaxone-resistant *Neisseria gonorrhoeae* strains identified in routine surveillance in Bangkok, Thailand, using Nanopore Q20+ chemistry, VolTRAX V2b, and Illumina sequencing

**DOI:** 10.1128/mra.01231-23

**Published:** 2024-02-01

**Authors:** Pongsathorn Sangprasert, Daniel Golparian, Porntip Paopang, Natnaree Girdthep, Ratana Lawung, Deyer Gopinath, Panithee Thammawijaya, Rossaphorn Kittiyaowanarn, Magnus Unemo

**Affiliations:** 1 Division of AIDS and STIs, Department of Disease Control and Prevention, Bangrak STIs Center, Thailand Ministry of Public Health, Bangkok, Thailand; 2 Department of Laboratory Medicine, Microbiology, Faculty of Medicine and Health, World Health Organization (WHO) Collaborating Centre for Gonorrhoea and Other Sexually Transmitted Infections, Örebro University, Örebro, Sweden; 3 Department of Clinical Microbiology and Applied Technology, Faculty of Medical Technology, Mahidol University, Bangkok, Thailand; 4 World Health Organization (WHO), Country Office, Nonthaburi, Thailand; 5 Department of Disease Control, Bangkok, Thailand; 6 Institute for Global Health, University College London (UCL), London, United Kingdom; Indiana University, Bloomington, Bloomington, Indiana, USA

**Keywords:** *Neisseria gonorrhoeae*, ceftriaxone resistance, Thailand

## Abstract

Ceftriaxone-resistant *Neisseria gonorrhoeae* strains, mostly associated with Asia, threaten gonorrhea treatment. We report the reference genomes of two ceftriaxone-resistant isolates found in routine surveillance in Bangkok, Thailand. The genomes belonged to the more antimicrobial-susceptible genomic lineage B, illustrating that both ceftriaxone-resistant strains and the mosaic *penA*-60.001 ceftriaxone-resistance determinant are spreading.

## ANNOUNCEMENT

Many *Neisseria gonorrhoeae* strains resistant to ceftriaxone, last-remaining gonorrhea treatment option, were identified in Europe, but nearly all were linked to Asia and several to Thailand (1–3). We report the complete reference genomes of two ceftriaxone-resistant gonococcal isolates (TH1713,TH2288) identified in the routine surveillance in Bangkok, Thailand.

The isolates were bedside cultured (diagnostic culture and for DNA extraction) on selective Modified Thayer-Martin Agar Medium (36 °C ±  1°C, 5% CO_2_, 24–48 h) from endocervical swabs of two Thai women attending Bangrak STIs Center in 2023 and verified as gonococci using MALDI-TOF MS (Bruker). Genomic DNA was extracted for all sequencing using the Nanobind CBB Big DNA Kit (Circulomics). Nanopore (Oxford Nanopore Technologies) libraries were prepared on a VolTRAX V2b (Nanopore) using the VolTRAX Sequencing Kit (VSK-VSK004), which includes enzymatic fragmentation without size selection, and sequenced on an Mk1C using R9.4.1 flowcells (FLO-MIN106D). Bases were called with guppy_basecaller (version 6.4.2) generating 7,642 (average Q-score: 22; average length: 9,282 bp) and 37,538 (average Q-score: 18; average length: 11,788 bp) reads for TH1713 and TH2288, respectively. Adapters were trimmed using Porechop (v0.2.4; https://github.com/rrwick/Porechop). Illumina DNA Prep (https://www.illumina.com/products/by-type/sequencing-kits/library-prep-kits/illumina-dna-prep.html) and NextSeq 550 platform (Illumina) were used for paired-end (150 bp) short-read sequencing [average depth: TH1713 517× (7,677,736 reads) and TH2288 98.6× (1,901,445 reads)]. Quality controls using CLC Genomics Workbench (v23.0.5) were performed to identify base quality with default parameters and trim according to quality score-limit Q30 using the CLC Trim Reads tool. K-mer spectra with WHO reference strains (*n* = 16) and K-mer length 16 (prefix ATGAC) were used for species confirmation and contamination control (4). Hybrid assemblies using Nanopore and Illumina reads were generated with Unicyler (v0.4.7) (5), including rotating the single contig to start with DnaA (UniRef: Q9JXS7). Circlator (v1.5.5) (6) rotated the plasmids to start with the replication initiator proteins for the cryptic plasmid (GenBank: CP116344.2) and conjugative plasmid (GenBank: CP116343.1). Default parameters were used except where otherwise noted.

The main characteristics of TH1713 and TH2288 and their complete reference genomes have been summarized in Table 1. Complete chromosomes of TH1713 and TH2288 were 2,228,256 bp and 2,227,834 bp, respectively, with 52.4% G+C content. Prokaryotic Genome Annotation Pipeline (PGAP, v6.1) (7) annotated 2,073 and 2,077 coding genes for TH1713 and TH2288, respectively. Both genomes contained sequences encoding 55 tRNA and 12 rRNA and harbored conjugative (39,060 bp; Dutch plasmid type) and cryptic (4,207 bp) plasmids. Phylogenomic analysis, performed as previously described (8), of 38,823 gonococcal genomes showed that both isolates belong to gonococcal genomic lineage B consisting mainly of isolates susceptible to currently used antimicrobials (4, 8, 9). The TH1713 and TH2288 genomes contained antimicrobial resistance determinants for ceftriaxone and ciprofloxacin, i.e., mosaic *penA*-60.001 and *gyrA* S91F/D95G, respectively. Both isolates had identical genotypes (Table 1), and pairwise distance calculation shows that the isolates were highly related with only 27 single nucleotide polymorphism (SNP) differences (https://pathogen.watch/collection/bx7vedyhw7v7-th1713-th2288). However, they were very distant from the internationally spreading ceftriaxone-resistant FC428 (3) clade (>5,000 SNPs), and the first described high-level ceftriaxone-resistant gonococcal strains [WHO X and Y (10)], all belonging to gonococcal genomic lineage A. Only one ceftriaxone-resistant isolate with mosaic *penA*-60.001 has been previously described in lineage B (8).

**TABLE 1:** The main characteristics of the ceftriaxone-resistant TH1713 and TH2288 gonococcal isolates cultured in Bangkok, Thailand, in 2023 and their complete reference genomes^*b*^

Antimicrobial susceptibility	TH1713	TH2288
Strain genotype		
Ceftriaxone (MIC)^*a*^	0.25	0.25
Cefixime (MIC)^*a*^	1	1
Azithromycin (MIC)^*a*^	0.25	0.25
Ciprofloxacin (MIC)^*a*^	2	2
*penA* allele	*penA-60.001*	*penA-60.001*
PorB1b G120, A121	WT	WT
*mtrR* promoter region 13 bp inverted repeat	WT	WT
*mtrR* coding region	WT	WT
Mosaic MtrRCDE	–	–
23S rRNA	WT	WT
Gyr A S91, D95	S91F, D95G	S91F, D95G
*rpsJ* V57	V57M	V57M
*tetM*	–	–
β-lactamase plasmid	–	–
NG-STAR	ST4320	ST4320
NG-STAR CC	CC1771	CC1771
MLST	ST8143	ST8143

^*a*^ MICs (mg/L) were determined using Etest (bioMérieux, Marcy-l’Etoile, France) for ceftriaxone, cefixime, azithromycin, and ciprofloxacin.

^*b*^ MIC, minimum inhibitory concentration; WT, wild type; NA, not applicable; NG-MAST, *Neisseria gonorrhoeae* multiantigen sequence typing; ST, sequence type; NG-STAR, *N. gonorrhoeae* Sequence Typing Antimicrobial Resistance; CC, clonal complex; MLST, multi-locus sequence typing.

## Data Availability

The complete chromosome including plasmids for TH1713 (CP138342, CP138343, CP138344) and TH2288 (CP138339, CP138340, CP138341) was deposited in GenBank. Raw Illumina (SRX22414127, SRX22414128) and Nanopore (SRX2241412, SRX22414130) reads are available through NCBI Short Read Archive. Project summary is found under BioProject PRJNA1036832.
